# Vulvar squamous cell carcinoma aggressiveness is associated with differential expression of collagen and STAT1

**DOI:** 10.1186/s12014-017-9175-8

**Published:** 2017-12-06

**Authors:** Emily R. Holthoff, Stephanie D. Byrum, Samuel G. Mackintosh, Thomas Kelly, Alan J. Tackett, Charles M. Quick, Steven R. Post

**Affiliations:** 1 Department of Pathology, University of Arkansas for Medical Sciences, 4301 West Markham Street, Slot 845, Little Rock, AR 72205 USA; 2 Department of Biochemistry and Molecular Biology, University of Arkansas for Medical Sciences, 4301 West Markham Street, Slot 845, Little Rock, AR 72205 USA

**Keywords:** Cancer, Cytokine, Cell adhesion, Proteomics, Stroma, Immune, Microenvironment, Pathognomic

## Abstract

**Background:**

Vulvar squamous cell carcinoma (vSCC) is a rare but debilitating disease. One vSCC variant comprises tumor cells that grow and expand as a cohesive sheet of cells that “pushes” and compresses the associated lymphoplasmacytic (LPC) stroma. Another vSCC variant features tumor cells that grow in loose association with one another and infiltrate the associated fibromyxoid (FMX) stroma consisting mainly of extracellular matrix. Clinically, infiltrative vSCC with FMX stroma (Inf/FMX) is significantly associated with lymph node metastases and recurrence.

**Methods:**

An unbiased proteomic approach was used to identify pathways involved in the development of the different vSCC variants. Proteins extracted from formalin-fixed and paraffin-embedded tissues of 10 cases of pushing vSCC with LPC stroma (Push/LPC) and eight cases of Inf/FMX were subjected to liquid chromatography-tandem mass spectrometry (LC–MS/MS).

**Results:**

Analysis identified 2265 different proteins in the 18 samples of vSCC. Of these, 282 proteins were differentially expressed between vSCC variants. Of these, 45 were higher and 237 lower in Inf/FMX compared to Push/LPC tumors. Consistent with the desmoplastic morphology and increased picrosirius red staining, expression of subunits of several collagens (Col 1, 3, 6, 14) was higher in the more aggressive Inf/FMX tumors. In contrast, signal transducer and activator of transcription 1 (STAT1), an important regulator of several inflammatory pathways, was expressed at lower levels in the Inf/FMX tumors. This finding was confirmed by immunohistochemistry using an antibody to STAT1. Informatics analysis of the differing profiles identified differences in pathways associated with integrin signaling and inflammation mediated by chemokines and cytokines.

**Conclusions:**

Comparing the proteomic profiles of vSCC morphologic variants indicates that increased expression of collagen subunits and decreased expression of STAT1 are associated with a more aggressive tumor variant, defined by increased incidence of nodal metastases and tumor recurrence. Informatic analyses further identify that both alterations in cell interaction with matrix and immune function differ with tumor aggressiveness. Identification of these pathways provides a molecular basis for understanding aggressiveness of vSCC.

**Electronic supplementary material:**

The online version of this article (10.1186/s12014-017-9175-8) contains supplementary material, which is available to authorized users.

## Background

Vulvar squamous cell carcinoma (vSCC) is a relatively rare cancer that is associated with a high rate of recurrence. The staging system for patients with vSCC is based on tumor size (as measured clinically), depth of invasion, extent of regional involvement, and lymph node status [1]. Treatment of vSCC patients based on the use of this staging system for prognostic stratification is associated with recurrence rates as high as 50–70% [2, 3]. Therefore, we opted to focus case selection based on our previously published findings which indicate that specific morphologic features reflective of differences in the tumor microenvironment are superior predictors of recurrence and tumor aggressiveness [4–6]. Specifically, tumors with an infiltrative tumor morphology and a fibromyxoid stroma (Inf/FMX) behaved more aggressively with a higher prevalence of tumor recurrence, perineural invasion (PNI), and nodal metastasis when compared to more indolent tumors having a pushing morphology with a lymphoplasmacytic stroma (Push/LPC) [4–6]. The association of these pathognomonic features with clinical outcomes indicates a fundamental difference in tumor–stroma interactions underlying the development of these vSCC variants.

Proteomic studies can be used to identify proteins that are specific to a disease (biomarkers) and that represent potential therapeutic targets for treating disease. Protein composition from different samples or groups of samples can be compared using bioinformatics approaches to identify altered protein expression and novel biomarkers of disease states. In the context of cancer, proteomic approaches have been used to identify the molecular interactions taking place within the tumor microenvironment and to highlight specific pathways that are driven by both host and tumor response [7, 8]. As an example, mass spectrometry was used to analyze protein expression in osteosarcoma biopsy samples and showed that patients whose biopsies contained higher expression of peroxiredoxin-2 (PRDX2) were less likely to respond to initial chemotherapy treatments [9]. These results were validated by demonstrating that reduced expression of PRDX2 in osteosarcoma cells lines increased response to various chemotherapeutic agents, indicating that PRDX2 is an important prognostic biomarker in patients with osteosarcoma.

In addition to identifying potential biomarkers, the functions of proteins and interactions between differentially expressed proteins can be analyzed using numerous databases and protein pathway analysis tools. Gene ontology (GO) assessment identifies molecular, cellular, and biological processes regulated by proteins of interest [10]. A recent study of samples from patients with bladder ischemia used GO assessment to show that many of the differentially expressed proteins were involved in proteolysis and other enzymatic processes thereby implicating protein degradation as an important step in chronic bladder ischemia [11]. A further analysis of signaling pathways indicated that many proteins differentially expressed in bladder ischemia samples are components of ERK/MAPK and ubiquitination signaling pathways and are associated with cellular damage and degeneration [11]. A similar proteomics workflow was used to study the differential protein expression between primary melanoma and metastatic melanoma formalin-fixed and paraffin-embedded (FFPE) samples. Proteins significantly altered in the metastatic lesions were associated with pathways linked to cancer progression using Pathway-Express (Onto-Tools) pathway analyses, providing important information about the progression of melanoma [12]. These examples highlight how identifying differential protein expression and altered molecular functions in tissue samples can provide intriguing and useful information about the molecular features of various disease states.

By defining proteins that comprise the tumor and its microenvironment, a proteomics–bioinformatics workflow provides an unbiased molecular approach to study differences in the tumor microenvironment and the functional roles of tumor–stromal interactions. Therefore, the objective of this study was to develop proteomic profiles that identify molecular differences between the Inf/FMX and Push/LPC variants of vSCC and to identify pathways that distinguish aggressive vSCC from indolent vSCC.

## Methods

### Case acquisition

Approval for research using archived human samples was obtained from the Institutional Review Board of the University of Arkansas for Medical Sciences (UAMS). We previously identified 143 cases of vSCC in UAMS case archives [4–6] and classified variants of these vSCCs as containing “pushing tumor” with lymphoplasmacytic stroma (LPC) or “infiltrative tumor” with fibromyxoid stroma (FMX) [5]. Due to the close association of nodal metastases and tumor recurrence with these morphologic variants [5], the current study used 10 cases of Push/LPC tumors and 8 cases of Inf/FMX tumors for proteomic analysis (Table 1). In addition, a section from a nondysplastic vulvar biopsy was used as control vulvar epithelial tissue for immunohistochemistry.

**Table 1:** Classification of vSCC cases

Sample nos.	Overall tumor morphology	Predominant stromal response	Age	Race
1	Pushing	LPC	60	C
2	Infiltrative	FMX	67	C
3	Pushing	LPC	65	C
4	Mixed	LPC	46	C
5	Infiltrative	FMX	61	C
6	Infiltrative	FMX	82	C
7	Pushing	LPC	52	B
8	Infiltrative	FMX	72	C
9	Pushing	LPC	38	C
10	Pushing	LPC	61	C
11	Pushing	LPC	60	C
12	Pushing	LPC	59	C
13	Infiltrative	FMX	53	C
14	Pushing	LPC	61	C
15	Pushing	LPC	80	C
16	Infiltrative	FMX	87	C
17	Infiltrative	FMX	67	C
18	Infiltrative	FMX	52	B

*LPC* lymphoplasmacytic, *FMX* fibromyxoid, *C* Caucasian, *B* Black

### Tissue processing

For each case, a 10 µm section of FFPE was adhered, but not heat-fixed, to a glass slide. Tissue proteins were extracted using 1.0 µL of 2% SDS harvest buffer for every 1.5 mm^2^ of tissue. Tissues were covered in harvest buffer, scored with a 20-gauge needle until solubilized into a gelatinous form, transferred into tubes, incubated at 90 °C for 30 min, and then sonicated (Bioruptor^®^ UCD200 ultrasonicator, Diagenode, Denville, NJ, USA) on high (200 W) for 15 min with 30 s on/off intervals. To reverse crosslinking, samples were incubated in the harvest buffer in a heating block at 65 °C overnight [13].

### SDS-PAGE and protein digestion

A modified Lowry protein assay was performed on each protein lysate using Bio-Rad *DC* Protein Assay reagents (Bio-Rad Laboratories, Inc., Hercules, CA, USA). Based on this protein assay, appropriate volumes needed for gel electrophoresis were calculated. Reducing agent, beta-mercaptoethanol (BME), was added to each protein extract, and samples were incubated at 90 °C for 5 min. Four micrograms of each sample and SeeBlue pre-stained protein standard (Novex, Thermo Fisher Scientific, Waltham, MA, USA) were loaded onto a pre-cast 1.0 mm 4–20% Tris/glycine gel (Novex) and electrophoresed at 125 V for 105 min. Gels were fixed in an acetic acid (10%) and methanol (16%) solution, stained with GelCode Blue Stain Reagent (Thermo Fisher Scientific), and imaged using a Kodak Image Station 4000 MM Pro.

### LC–MS/MS and bioinformatic analysis

Each sample gel lane was cut into 20 2-mm sections and subjected to in-gel trypsin digestion as described previously [14]. Briefly, gel slices were destained in 50% methanol (Fisher, Thermo Fisher Scientific), 100 mM ammonium bicarbonate (Sigma-Aldrich, Merck Group, St. Louis, MO, USA), followed by reduction in 10 mM Tris[2-carboxyethyl]phosphine] (Pierce, Thermo Fisher Scientific) and alkylation in 50 mM iodoacetamide (Sigma-Aldrich). Gel slices were then dehydrated in acetonitrile (Fisher, Thermo Fisher Scientific), followed by addition of 100 ng porcine sequencing grade modified trypsin (Promega, Madison, WI, USA) in 100 mM ammonium bicarbonate (Sigma-Aldrich) and incubation at 37 °C for 12–16 h. Peptide products were then acidified in 0.1% formic acid (Pierce, Thermo Fisher Scientific).

Tryptic peptides were separated by reverse phase Jupiter Proteo resin (Phenomenex, Torrance, CA, USA) on a 100 × 0.075 mm column using a nanoAcquity UPLC system (Waters Corporation, Milford, MA, USA). Peptides were eluted using a 30 min gradient from 97:3 to 60:40 buffer A:B ratio [Buffer A = 0.1% formic acid, 0.5% acetonitrile; buffer B = 0.1% formic acid, 90% acetonitrile.] Eluted peptides were ionized by electrospray (1.9 kV) followed by MS/MS analysis using collision induced dissociation on an LTQ Orbitrap Velos mass spectrometer (Thermo Fisher Scientific). MS data were acquired using the FTMS analyzer in profile mode at a resolution of 60,000 over a range of 375–1500 *m/z*. MS/MS data were acquired for the top 15 peaks from each MS scan using the ion trap analyzer in centroid mode and normal mass range with a normalized collision energy of 35.0. The proteomics data was generated in the UAMS Proteomics Core.

Proteins were identified by searching the UniProtKB database (restricted to *Homo sapiens*, 157537 entries) using the Andromeda search engine in MaxQuant (version 1.5.3.8). The database was searched using a decoy database with the reverse sequences in order to calculate the false discovery rate, which was determined to be 1% [15]. Search parameters were as follows: trypsin digestion with up to three missed cleavages; fixed modification of carbamidomethyl of cysteine; variable modifications of oxidation on methionine and acetyl on N-terminus; first search set to 5 ppm precursor ion tolerance and the main search was set to 3 ppm; selected label-free quantitation with intensity-based absolute quantification (iBAQ) with a minimum ratio of 1. The peptide spectral match (PSM) and protein false discovery rates were set to 1%. A contaminants database (245 entries) was used for the first search to identify commonly identified contaminants. Data output from the MaxQuant analysis are provided in Additional file 1: Supplemental Table 1.

The 4 most abundant proteins (hemoglobin alpha and beta, keratin, and actin) were subtracted from the sum total intensity values because these proteins were in high abundance in all samples and are commonly identified from tumor samples. Individual protein intensities were then corrected to account for differences in overall protein mass between samples using a normalization factor calculated from the sample with the lowest sum iBAQ intensity. Missing intensity values (i.e., for proteins not identified in a particular sample) were replaced with the lowest protein intensity detected in any sample as a minimal threshold value to facilitate further analysis [16, 17]. The data were then log_2_ transformed for statistical analysis using the FDR method of Benjamini and Hochberg, with a Q value of 20 (FDR = 20%) in GraphPad Prism 6.0. Fold change of proteins was calculated by subtracting the average log_2_ normalized iBAQ of Inf/FMX from the log_2_ normalized iBAQ of Push/LPC [14].

Proteins that were differentially expressed (*p* < 0.05), had a FDR < 20% and a greater than fourfold difference between tumor variants were examined using hierarchical clustering with the average linkage method and Euclidean distance metric in the Hierarchical Clustering Explorer (HCE, version 3.5). The log_2 _normalized iBAQ data was standardized by the mean and standard deviation prior to performing the clustering algorithm for both the tumor samples and the proteins. Proteins that were significantly different between tumor variants were analyzed using the PANTHER functional classification online analysis tool (PANTHER™ Version 12.0; released 2017-07-10) [18–20], and Ingenuity^®^ Pathway Analysis Software (Qiagen Bioinformatics; version: 39480507; year: 2017) to identify important pathways.

### Special staining and IHC

For collagen staining, FFPE sections from each case were sectioned, deparaffinized, and then incubated sequentially with a solution of phosphomolybdic acid (0.2% w/v) and picrosirius red (0.1% w/v). After staining, digital images were captured with bright-field and polarized illumination.

For STAT1 immunostaining, FFPE from each case was sectioned and heat fixed to slides, deparaffinized, and subjected to citrate-based heat-induced epitope retrieval. Slides were then stained with a STAT1(42H3) rabbit monoclonal antibody (Cell Signaling, Danvers, MA, USA, product #9175; 1:500), followed by an anti-rabbit secondary antibody, and incubation with DAB. The stained slides were scanned using an Aperio ScanScope^®^ and analyzed for positive pixel count in a 10 × area of the tumor–stroma interface in the Aperio ImageScope^®^ program (Leica Biosystems, Wetzlar, Germany). The total number of positive pixels identified in a sample of nondysplastic vulvar epithelial tissue was subtracted from the total number of positive pixels in each of the vSCC cases. The corrected number of positive pixels for each case was then divided by the area of the tissue section to determine the pixel intensity per area of tissue. To ensure Gaussian assumption, IHC intensities were log transformed and compared using a *t* test.

## Results

**Fig. 1:**
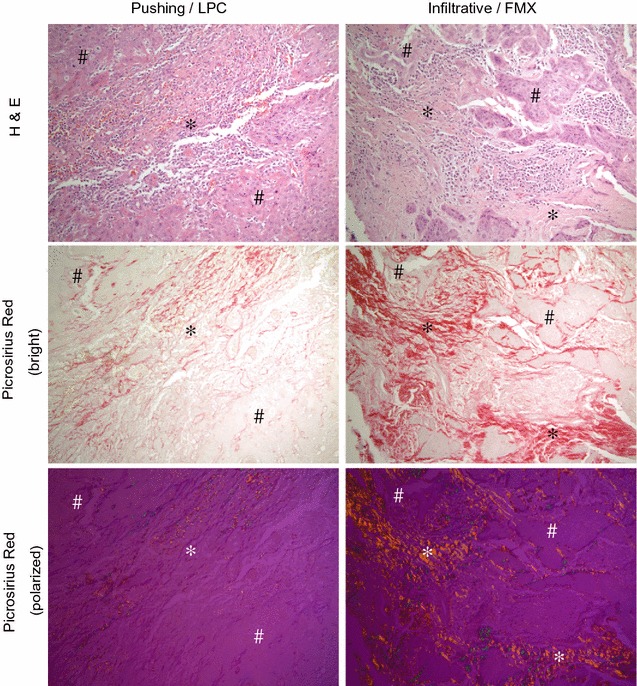
Patterns of tumor invasion in vSCC. H&E (top panels) and picrosirius red (middle and bottom panels) staining of Push/LPC (left panels) and Inf/FMX (right panels) of vSCC. Tumor areas are indicated with a ‘#’ and stromal areas with a ‘*’. Picrosirius red staining is shown in bright field (middle panels) where collagen is stained red, and in polarized illumination (bottom panels) where larger collagen fibers are stained yellow-orange and thinner fibers, including reticular fibers, appear green. Photographed with ×20 objective

Morphologic variants of vSCC have been described and correlated with tumor aggressiveness [4–6]. As depicted in Fig. 1, the more indolent of these variants is defined by a “pushing” tumor morphology characterized by a clearly demarcated border between sheets of invading tumor cells and an inflammatory lymphoplasmacytic (LPC) stroma. The more aggressive vSCC variant has an infiltrative tumor morphology comprised of cords and single tumor cells invading into a collagen-rich or fibromyxoid (FMX) stroma. Picrosirius red staining (Fig. 1) reveals relatively low collagen levels in the LPC stroma of pushing tumors as compared to abundant collagen that is highly organized in the FMX stroma of infiltrative tumors. Defining the molecular and functional differences between these morphologic variants provides an opportunity to understand the prognostic indicators of aggressive vSCC. In this study, a proteomic approach was used to provide unbiased insight into molecular variations associated with the pathognomonic features of vSCC.

**Fig. 2:**
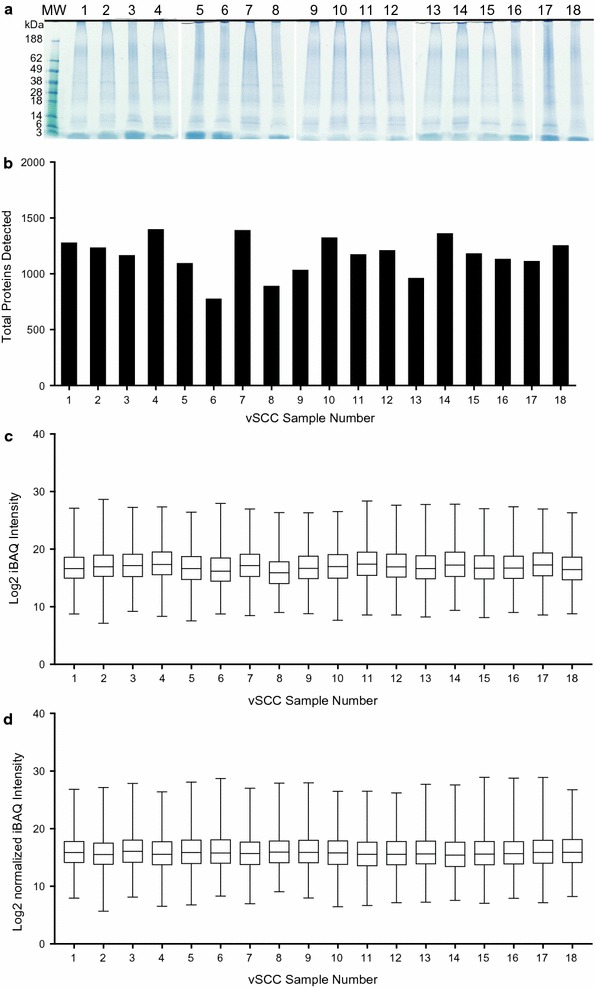
Proteomic analysis of vSCC samples. **a** vSCC samples (#1–18) were resolved with 4–20% Tris–Glycine gels and protein stained with Coomassie. Individual lanes represent protein extract from a single vSCC sample. **b** Proteins from individual samples were subjected to LC/MS/MS and iBAQ quantification, and the total number of distinct proteins identified in each vSCC sample was determined. **c**, **d** Box plots representing the total protein intensities determined for each sample following removal of the 4 most abundant protein intensities before (**c**) and after (**d**) normalization

Eighteen cases of vSCC (Table 1) were selected by pathologists based on characterization as Push/LPC (10 cases) or Inf/FMX stroma (eight cases). Proteins were extracted from FFPE sections and resolved by SDS-PAGE (Fig. 2a). Gel samples were cut into smaller pieces, in-gel digested with trypsin, and subjected to proteomic analysis. High resolution LC–MS/MS analysis of FFPE protein extracts resulted in identification of 2265 different proteins in the 18 samples of vSCC with a false discovery rate (FDR) of 1%. Of those, 400 proteins (17.66%) were detected in all eighteen samples. The total number of proteins identified and the iBAQ intensities before and after normalization for each sample are shown in Fig. 2b–d. The average number of proteins identified in individual samples was 1167 ± 170. The number of common proteins identified in at least one sample in each group is 1814 (Inf/FMX; 80%) and 2042 (Push/LPC; 90%). The number of common proteins identified in all of the samples in each group is 470 (Inf/FMX) and 594 (Push/LPC).

**Fig. 3:**
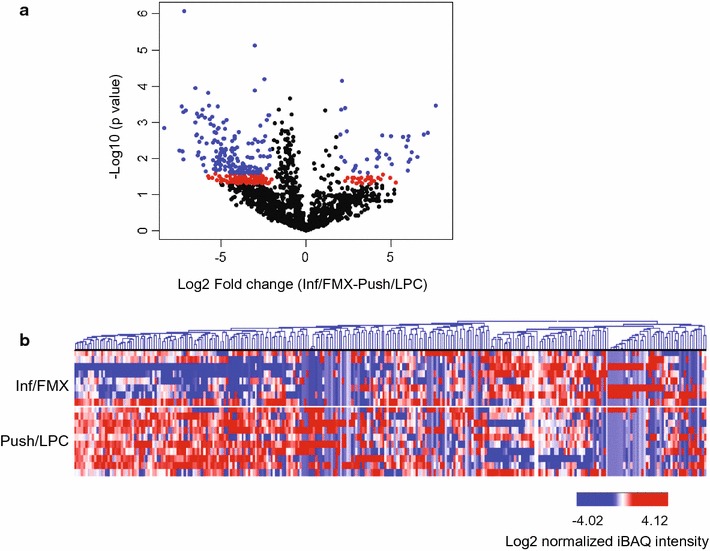
Differential protein expression in vSCC variants. **a** Volcano plot showing the 282 of 2265 proteins that were significantly (*p* < 0.05) different by at least fourfold (red and blue dots) between Inf/FMX and Push/LPC, and those with a FDR < 20% (blue dots only). Significant proteins and FDRs were determined using the FDR method of Benjamini and Hochberg. *p* < 0.05 was considered significant and is reflected by a − log_10_ value of 1.3. **b** Hierarchical clustering of log_2_-transformed intensity values shows the distribution of the 282 significantly different proteins between the two variants

The protein expression profiles of Inf/FMX and Push/LPC vSCC were compared using the FDR method of Benjamini and Hochberg, with a Q value of 0.20 (FDR = 20%) to determine differentially expressed proteins. There were 282 proteins that differed between the two tumor variants (Additional file 1: Supplemental Table 2). As depicted in a volcano plot (Fig. 3a), 45 proteins showed a higher level (35 were ≥ fourfold higher) and 237 a lower level of detection (174 were ≥ fourfold lower) in the Inf/FMX than in Push/LPC tumors. These distinct expression profiles between the tumor variants are also depicted in a heat map (Fig. 3b). The hierarchical cluster of both proteins and samples indicates the significant proteins are able to separate the Inf/FMX tumors from the Push/LPC tumors. Interestingly, 7 of the 45 (16%) proteins that were higher expressed in Inf/FMX tumors were subunits of collagen. This result is consistent with the abundance of collagen observed in this tumor variant (Fig. 1) and demonstrates the ability of the proteomic approach to identify relevant proteins in the vSCC samples.

**Fig. 4:**
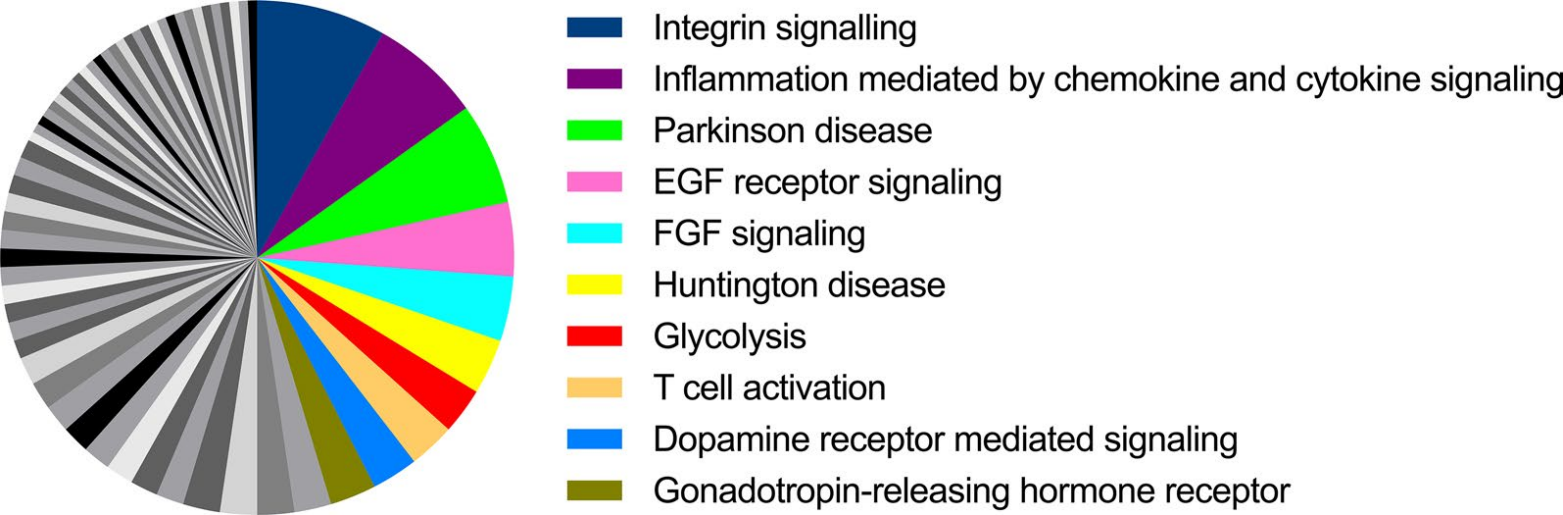
PANTHER identified pathways matching at least five components. Proteins with differing expression between vSCC variants were analyzed with PANTHER to identify pathways that differed. Shown is a pie chart representing the 64 different pathways with pathways associated with 5 or more significantly altered components highlighted. A detailed list of all pathways is available in Additional file 1: Supplemental Table 3

To identify molecular and cellular pathways that underlie the observed differences in expression of proteins between the two tumor variants, the proteins that were differentially expressed with an FDR ≤ 20% between tumor variants were examined using the PANTHER functional classification online analysis tool [18–20] and ingenuity pathway analysis (IPA). The PANTHER Pathway database includes over 177 pathways and performs an analysis of pathway components. A component pathway analysis in PANTHER of the 282 proteins identified 160 components matched to 64 different pathways (Fig. 4; Additional file 1: Supplemental Table 3). The pathways identified by PANTHER with the greatest number of identified pathway components were the integrin signaling pathway (14 genes, Table 2) and the inflammation mediated by chemokine and cytokine signaling pathway (12 genes, Table 3). The proteins associated with the most pathways were Rac (17 pathways), Grb2 (16 pathways) and STAT1 (9 pathways). Of these proteins, STAT1 showed the greatest difference in expression between tumor subtypes and was associated with inflammation mediated by chemokine and cytokine signaling pathway. Analysis with IPA identified 103 canonical pathways that were significantly different between tumors (Additional file 1: Supplemental Table 4). Similar to the PANTHER analysis, Grb2, Rac1 and STAT1 were the proteins most commonly associated with pathways identified by IPA. STAT1 was a component of seven pathways, most of which are involved in inflammatory immune responses (Table 4). Additional pathways that were identified with high significance include the EIF2, mTOR, p70S6K, and HIPPO signaling pathways.

**Table 2:** Matched components of the integrin signaling pathway

Mapped ID	Gene name (PANTHER family/subfamily)	Panther protein class
ACTG1	Actin, cytoplasmic 2	Actin, cytoplasmic 2 (PTHR11937:SF362)
ACTN2	Alpha-actinin-2 (PTHR43947:SF4)	
ARPC5L	Actin-related protein 2/3 complex subunit 5-like protein (PTHR12644:SF2)	Actin family cytoskeletal protein (PC00041)
CDC42	Cell division control protein 42 homolog (PTHR24072:SF136)	Small GTPase (PC00208)
COL14A1	Collagen alpha-1(XIV) chain (PTHR44557:SF1)	
COL1A1	Collagen alpha-1(I) chain (PTHR24023:SF569)	
COL1A2	Collagen alpha-2(I) chain (PTHR24023:SF568)	
COL3A1	Collagen alpha-1(III) chain (PTHR24023:SF604)	
COL6A1	Collagen alpha-1(VI) chain (PTHR44172:SF3)	
COL6A2	Collagen alpha-2(VI) chain (PTHR44172:SF1)	
COL6A3	Collagen alpha-3(VI) chain (PTHR44105:SF2)	
GRB2	Growth factor receptor-bound protein 2 (PTHR24418:SF290)	Non-receptor tyrosine protein kinase (PC00168)
LAMC1	Laminin subunit gamma-1 (PTHR10574:SF270)	Extracellular matrix linker protein (PC00101); receptor (PC00197)
RAC1	Ras-related C3 botulinum toxin substrate 1 (PTHR24072:SF105)	Small GTPase (PC00208)

**Table 3:** Matched components of the inflammation mediated by chemokine and cytokine signaling pathway

Mapped IDs	PANTHER family/subfamily	Panther protein class
ACTG1	Actin, cytoplasmic 2 (PTHR11937:SF362)	Actin and actin related protein (PC00039)
ARPC5L	Actin-related protein 2/3 complex subunit 5-like protein (PTHR12644:SF2)	Actin family cytoskeletal protein (PC00041)
CDC42	Cell division control protein 42 homolog (PTHR24072:SF136)	Small GTPase (PC00208)
COL14A1	Collagen alpha-1(XIV) chain (PTHR44557:SF1)	
COL6A1	Collagen alpha-1(VI) chain (PTHR44172:SF3)	
COL6A2	Collagen alpha-2(VI) chain (PTHR44172:SF1)	
COL6A3	Collagen alpha-3(VI) chain (PTHR44105:SF2)	
GNAI3	Guanine nucleotide-binding protein G(k) subunit alpha (PTHR10218:SF230)	Heterotrimeric G-protein (PC00117)
GRB2	Growth factor receptor-bound protein 2 (PTHR24418:SF290)	Non-receptor tyrosine protein kinase (PC00168)
IL18	Interleukin-18 (PTHR45200:SF1)	
RAC1	Ras-related C3 botulinum toxin substrate 1 (PTHR24072:SF105)	Small GTPase (PC00208)
STAT1	Signal transducer and activator of transcription 1-alpha/beta (PTHR11801:SF18)	Nucleic acid binding (PC00171); transcription factor (PC00218)

**Table 4:** IPA identified canonical pathways associated with STAT1

1	UVA-induced MAPK signaling
2	Role of PKR in interferon induction and antiviral response
3	Production of nitric oxide and reactive oxygen species in macrophages
4	iNOS signaling
5	Hepatic fibrosis/hepatic stellate cell activation
6	Dendritic cell maturation
7	ERK/MAPK signaling

**Fig. 5:**
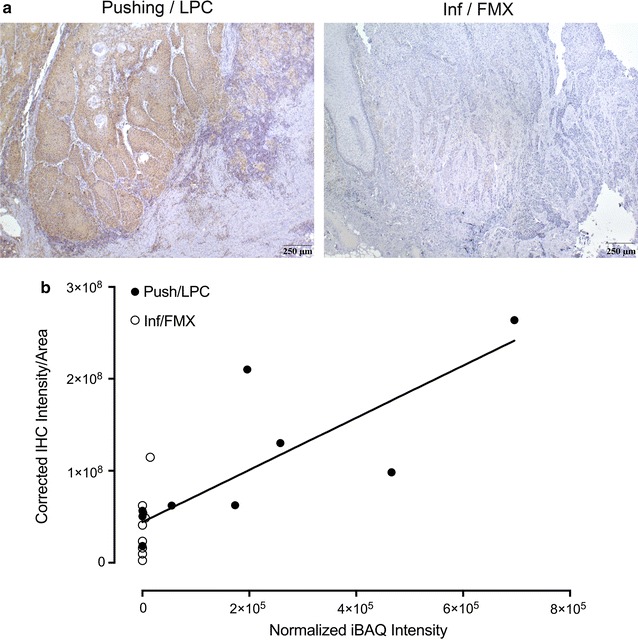
STAT1 IHC expression in vSCC. **a** Immunostaining of STAT1 in Push/LPC (left) and Inf/FMX (right) vSCC tumors. Tumors shown at ×10 magnification. **b** Intensities of STAT1 immunostaining were obtained with Aperio ImageScope^®^ program and calculated as number of positive pixels per area of tissue after subtracting non-specific staining as determined using non-dysplastic tissue. The graph shows the linear regression analysis of normalized STAT1 iBAQ intensities detected by proteomic analysis and corrected IHC immunostaining intensities for STAT1

It is important to validate the results of the proteomics and bioinformatics with the observed pathology in vSCC. The increased expression of collagen subunits detected by proteomics and identified as major components in the integrin signaling pathway is evident by the picrosirius red staining of tumor sections (Fig. 1). Because an important pathognomonic feature that distinguishes the Inf/FMX and Push/LPC vSCC subtypes is the stromal immune response, the changes in STAT1 protein detected in the proteomic analysis were examined by IHC. FFPE sections for each of the 18 cases were stained with antibody to STAT1. As shown in Fig. 5a, STAT1 staining was strong to moderate in both tumor and stromal regions in the majority of Push/LPC tumors (left panel); whereas, most of the Inf/FMX tumors exhibited mild to absent staining of STAT1(right panel). A linear regression analysis was then performed to determine the correlation between IHC staining intensity (pixel intensity per area of tissue) and normalized iBAQ intensities for STAT1 from each protein sample. There was a significant increase in STAT1 staining in Push/LPC relative to Inf/FMX tumors (2.53-fold, *p* = 0.048). A linear regression model showed a significant correlation (*R* ^2^ = 0.64; *p* < 0.0001) between STAT1 IHC intensity and STAT1 iBAQ intensity (Fig. 5b), indicating that the data obtained from the proteomic analysis is an accurate reflection of STAT1 expression in these 18 tumors.

To begin to understand how STAT1 might be involved in vSCC progression, an upstream regulator analysis, which uses a z-score algorithm to make predictions, was performed in IPA using the differentially expressed proteins. This analysis identified STAT1 as an upstream regulator of several proteins that showed decreased expression in the Inf/FMX tumors and were predicted to be associated with decreased STAT1 activity. These proteins include the proteasome activator subunits 1 and 2 (PSME1, PSME2), the interferon-gamma induced protein 16 (IFI16), beta-2-microglobulin (B2M), CD14, lipocalin 2 (LCN2), and ISG15 ubiquitin-like modifier (ISG15). Each of these proteins is associated with regulating immune function, and their lower expression in Inf/FMX tumors is consistent with decreased STAT1 expression.

## Discussion

In this study, two major morphologic variants of vSCC, which are associated with differing prognostic implications, were examined using a proteomic approach to better understand the molecular and cellular processes that generate these distinct tumor–stroma variants. The data indicate that the more aggressive infiltrative vSCC with FMX stroma have increased collagen relative to the pushing vSCC with LPC stroma. This finding is consistent with the desmoplastic morphology and picrosirius red staining of this variant. Further analysis reveals that STAT1 is lower in the Inf/FMX tumors. This suggests that pathways in which STAT1 participates are important to driving the LPC response in pushing vSCC and perhaps explaining the absence of such an inflammatory response in the infiltrative tumors.

Using PANTHER and IPA to assess the functional classifications and signaling pathways associated with the 282 proteins that significantly differed between Inf/FMX and Push/LPC tumors identified signaling pathways associated with integrins and inflammation mediated by chemokines and cytokines as those associated with the most protein alterations. In large part, this reflected the significantly higher expression of multiple collagen subunits and lower expression of STAT1 in the more aggressive Inf/FMX variant. In addition, proteins associated with cell–matrix adhesion were overrepresented in the Inf/FMX variant; whereas proteins associated with immune function and fibrosis were overrepresented in the Push/LPC variant. Together, these analyses highlight the importance of the stromal response and the interactions occurring within the tumor microenvironment in determining tumor aggressiveness.

The abundance of collagen found in the fibromyxoid stroma likely has an active role in promoting the aggressiveness of the infiltrative vSCC tumors that have a poorer prognosis than pushing vSCC. First, increased stiffness of the extracellular matrix is known to stimulate invasive behavior of tumor cells [21], and collagens have been shown to contribute to increased stiffness of the matrix [22]. Such stiffness in human prostate cancer xenografts can be measured by 2-dimensional sonography and shear wave elasticity [22]. In addition to changing the physical properties of the microenvironment, increased collagens contribute to immune suppression. For example, weaning-induced breast involution has been shown to result in a collagen-rich and immune-suppressed microenvironment that is tumor promotional in mice [23]. Finally, as previously described, the tumor-supportive desmoplastic stromal response seen in cancers is similar to the body’s attempt at wound healing [24–26]. The FMX stroma seen in infiltrative vSCC mirrors the wound healing environment both at the structural level, with an abundance of tissue-remodeling fibroblasts and collagen, and at the proteomic level.

**Fig. 6:**
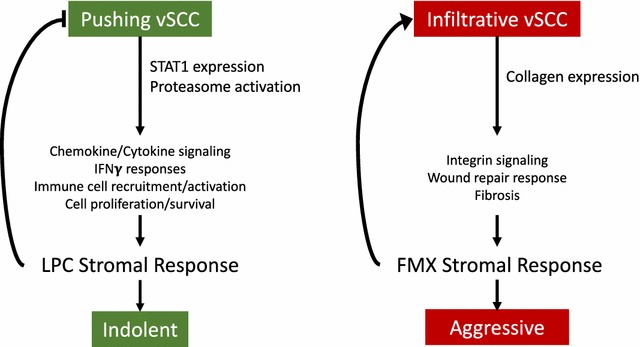
Proposed role of observed changes in protein levels in vSCC variants. As determined by proteomic profiling, vSCC tumors with a pushing morphology are associated with increased expression of STAT1 and proteasome proteins. As a consequence, there is activation of a signaling pathways associated with a lymphoplasmacytic stromal response, which inhibits tumor growth and aggressive behavior. In contrast, tumors with an infiltrative morphology have increased collagen expression with activation of integrin signaling resulting in the desmoplasia that defines a fibromyxoid stromal response. In the absence of a strong immune response, this promotes tumor growth and aggressive behavior

STAT1 is an intracellular protein that modulates various cellular processes by delivering transmembrane signals from cytokines, interferons, and some interleukins to the nucleus. STAT1 plays a pivotal role in both innate and adaptive immune responses, and regulates the transcription of various proteins [27, 28]. Importantly, comparison of the two vSCC variants with respect to tumor recurrence, nodal metastasis, and PNI consistently reflected the importance of the immune response as a major feature that distinguishes more aggressive (little or no immune response) from more indolent tumor phenotypes (vigorous immune infiltrate) [4–6]. This data suggests that the absence of an inflammatory stromal response in infiltrative tumors may be due to a decrease in STAT1 signaling while in pushing tumors, the LPC stroma is undergoing a host immune reaction mediated, in part, by STAT1. Consistent with this interpretation, IPA identified several pathways related to cell proliferation and survival (e.g., mTOR, p70S6K, protein ubiquitination). Additional pathways that are regulated by STAT1 were related to the function of macrophages/dendritic cells and tissue fibrosis (Table 4). Changes in the expression of STAT1 and downstream proteins associated with immune response and proteasome function suggests a possible mechanism for regulating the differential stromal cell responses and clinical outcomes observed in vSCC (Fig. 6). These interactions make STAT1 an extremely intriguing protein to study in the vSCC cohort.

STAT1 has been considered a tumor suppressor through mechanisms of increased anti-tumor immune response [27, 29], and STAT1 expression has been associated with improved outcomes in cancer [30–32]. However, some studies have shown that STAT1 may be linked to tumorigenesis, decreased response to therapy, and overall poorer outcomes for cancer patients [33–35]. A previous proteomic study of vSCC classified samples based on patient HPV status and associated decreased STAT1 expression and proteosomal proteins with HPV infection and lower incidence of relapse [36]. Interestingly, we also detected alterations in the expression of STAT1 and proteosomal proteins; however, we found very low levels of STAT1 and decreased proteosomal proteins (PSME1/2) in the Inf/FMX variant which is associated with a high incidence of tumor recurrence and nodal metastasis [5]. The reason for this difference is not clear, but suggests that the aggressive behavior of the Inf/FMX variant is independent of STAT1, and involves upregulation of collagens with a notable absence of host immune response (Fig. 6). Although the specific contributions of STAT1 to vSCC progression have not been fully elucidated, it is clear that this protein is important in tumor-initiated immune responses.

## Conclusions

Comparing the proteomic profiles of vSCC variants indicates that higher expression of collagen subunits and lower expression of STAT1 are associated with a more aggressive vSCC variant that is characterized by an infiltrative tumor morphology and a fibromyxoid stromal response. Informatic analyses of the different proteomic profiles further associate both the alterations in cell interaction with matrix and the immune function with tumor aggressiveness. Identification of these pathways suggests that a collagen-rich and immune-suppressed microenvironment promotes aggressiveness of vSCC.

## Additional file

**Additional file 1: Table S1.** iBAQ intensities. **Table S2**: Proteins differentially expressed in vSCC variants. **Table S3**: PANTHER pathway analysis of proteins differentially expressed in vSCC variants. **Table S4**: Canonical pathways identified by IPA.
